# Dr. Stanley Emanuele Tron

**Published:** 1913-09

**Authors:** 


Dr. Stanley Emanuele Tron, Harvard, 1910, who had been located in Utica for several months, died June 22, aged 29, of morphine poisoning. The first reports ascribed his death to suicide, but more recently suspicion has been aroused that he was murdered.

